# SARS-CoV-2-Associated Multisystem Inflammatory Syndrome in Children (MIS-C): A Case Report from Iraq

**DOI:** 10.3390/pediatric15030048

**Published:** 2023-09-04

**Authors:** Ruwaid Behnam Y. Al-Simaani, Lika’a Fasih Y. Al-Kzayer, Kenan Hussien Ali, Mouroge H. Al-Ani, Yozo Nakazawa

**Affiliations:** 1 Department of Pediatrics, Al-Sheikhan General Hospital, Nineveh 42012, Iraq; 2 Department of Pediatrics, Noorjan Medical Center, Erbil 44001, Iraq; 3 Department of Pediatrics, Shinshu University School of Medicine, Matsumoto 390-8621, Japan; 4 Department of Family Medicine, College of Medicine, Baghdad University, Baghdad 10071, Iraq; 5 Department of Pediatrics, Nana-Kali Hospital for Hemato-Oncology, Erbil 44001, Iraq

**Keywords:** MIS-C, COVID-19, SARS-CoV-2, Iraq

## Abstract

The novel coronavirus disease (COVID-19) continues to evolve. Severe acute respiratory syndrome coronavirus-2 (SARS-CoV-2)-associated multisystem inflammatory syndrome in children (MIS-C) is a rare post-COVID-19 complication that affects children with critical outcomes. Few MIS-C reports were available from Arab-Asian ethnicities. We here describe a presentation mimicking a head injury overlapping the manifestations of MIS-C in a child from Iraq. A 10-year-old boy presented with blunt trauma in a shock-like status, and a head injury was suspected. Since he was febrile two days before the trauma, another pathology was assumed. Imaging and laboratory evaluations were performed, and after excluding gross neurosurgical etiology, he was initially treated as a toxic shock syndrome. Meanwhile, he was deteriorating with continuous fever, impaired consciousness, and seizure on the following day. Although not considered initially, close monitoring with a multidisciplinary approach and serial investigations revealed that the child met the criteria of MIS-C. SARS-CoV-2 IgG was shown to be high, while the RT-PCR of COVID-19 was negative. Once he received immunoglobulin and methylprednisolone, he improved dramatically. In conclusion, this report aimed to increase awareness about MIS-C among health workers and emphasized the need for a multidisciplinary team approach in Iraq due to the importance of timely treatment.

## 1. Introduction

A novel coronavirus disease-2019 (COVID-19) was identified at the end of 2019 and rapidly reached a pandemic level in accordance with World Health Organization (WHO) definition. COVID-19 is caused by the severe acute respiratory syndrome coronavirus 2 (SARS-CoV-2) [1]. SARS-CoV-2-associated multisystem inflammatory syndrome in children (MIS-C) is a rare post-COVID-19 complication, occurring 2–6 weeks after COVID-19. MIS-C commonly affects the pediatric age group with substantial morbidity and is potentially life threatening [2,3,4]. COVID-19 infection among children is usually mild, and the absence of confirmation tests is often expected. However, a severe infection could also happen in children. Therefore, MIS-C incidence is unclear, and it was shown to occur in 3/1000 (<1%) of children with confirmed COVID-19 infection [3,4]. MIS-C is a multi-organ illness similar to Kawasaki disease or toxic shock syndrome features, albeit not in all findings. Apart from fever and rising markers of inflammation, MIS-C can affect several organs, including the gastrointestinal, mucocutaneous, cardiovascular, coagulation, neurocognitive, and respiratory systems [3,4,5,6]. According to ongoing studies, the mechanism behind MIS-C is probably attributed to an exaggerated immune dysregulation after COVID-19 [5]. To diagnose MIS-C, several criteria defined by the Centers for Disease Control and Prevention (CDC) and WHO must be fulfilled [5,7].

Of note, few cases of MIS-C were available from Arab-Asian ethnicities, including Iraq [4]. Iraq is an Asian country where the majority of the population is of Arab ethnicity, and 60% of its population is younger than 25 years old. Iraq was one of the countries heavily affected by the prevalence of COVID-19, with a much higher number of deaths compared to other Mediterranean countries. However, publications concerned with COVID-19 from Iraq are remarkably few [8]. One article that enrolled 31 children with criteria of MIS-C from the Kurdistan Region inhabited by the Kurdish ethnicity was found [9].

## 2. Case Description

**Figure 1:**
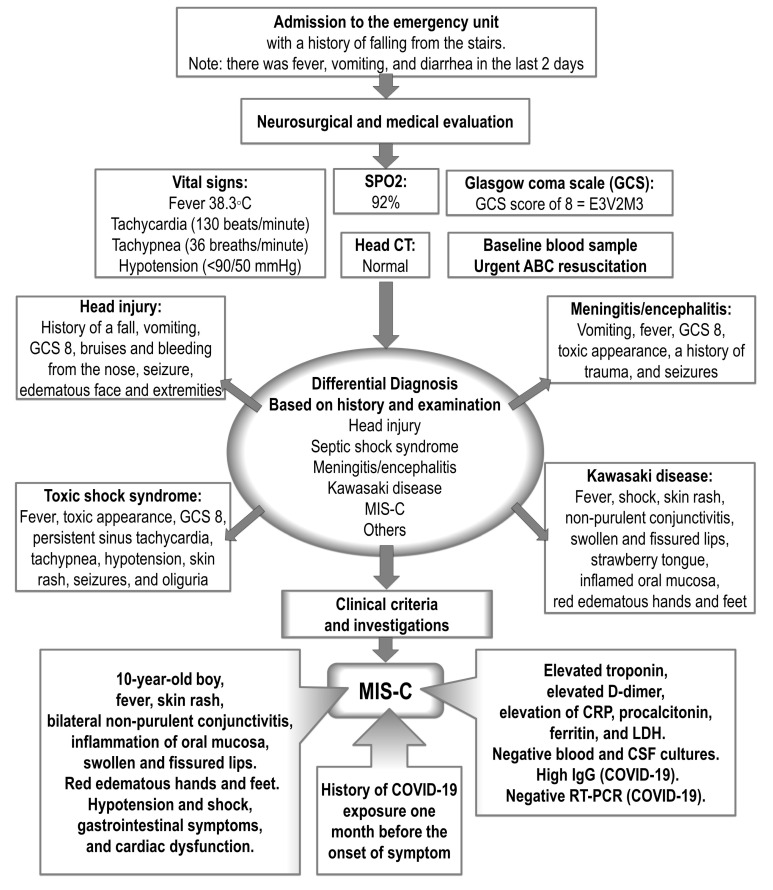
A diagram of the approach to the case of multisystem inflammatory syndrome in children (MIS-C) presented after blunt trauma. GCS, Glasgow coma scale; (GCS = 8/15); (E), eye; (V), verbal; (M), motor; CT, computed tomography; COVID-19, coronavirus disease; CRP, c-reactive protein; LDH, lactate dehydrogenase; CSF, cerebrospinal fluid examination; RT-PCR, reverse transcription polymerase chain reaction.

On 12 March 2022, a 10-year-old boy of Arab-Asian ethnicity from Mosul, Iraq, fell from the stairs and got a nosebleed. His condition deteriorated, with headache, dizziness, nausea, vomiting, and altered consciousness. He was seen at the emergency department of a general hospital in Erbil with the suspicion of a head injury. Providing that the patient’s family gave a history of fever, headache, vomiting, and diarrhea for 2 days before admission, another pathology was considered, and dizziness could be the cause of falling (Figure 1). Upon admission, the boy was looking toxic with impaired consciousness, his oxygen saturation (SPO2) was 92%, he was febrile (38.3 °C), had tachycardia (130 beats/min), tachypnea (36 breaths/min), and hypotension as his blood pressure was less than the 5th percentile (<90/50 mmHg). In addition, his face was edematous, with bruises over the nose and bleeding from the nose. He was unconscious with flexion to pain, and his Glasgow coma scale (GCS) scored 8/15 (Eye, 3; Verbal, 2; and Motor, 3). Supportive care was given to maintain oxygenation, ventilation, and circulation, with head elevation to 15 degrees, and cardiorespiratory monitoring was set. However, the ventilator was not required. Crystalloid intravenous (iv) fluid, antipyretics, and empiric antibiotics (vancomycin and ceftriaxone) were given while obtaining samples for laboratory tests. Urgent computed tomography (CT) of the head was arranged to exclude the fracture at the base of the skull and to determine if there is bleeding inside or around the brain or other serious brain injuries. A neurosurgeon was consulted, and the patient was referred to the intensive care unit (ICU). The CT was normal. The baseline investigations were conducted, including random blood sugar, blood gas analysis and electrolytes, c-reactive protein (CRP), complete blood count (CBC), blood culture, renal function test, and liver function test (LFT). LFT included lactate dehydrogenase (LDH) and gamma-glutamyl transferase (GGT), in addition to alanine aminotransferase (ALT) and aspartate aminotransferase (AST). Also, coagulation markers were evaluated to assess prothrombin time, international normalization ratio, activated thromboplastin time, and D-dimer.

Further investigations, such as troponin T and procalcitonin, were conducted later. Ultrasound (US) of the abdomen, portable chest X-ray (CXR), electrocardiography (ECG), and echocardiography were also performed. The concerning results are shown in Table 1.

The patient also had bilateral non-purulent conjunctivitis, and his pupils were dilated, reacting to light with normal fundoscopy. He had inflamed oral mucosa with swollen, dried, cracked lips, a strawberry tongue, and a skin rash. He also had red edematous hands and feet. His abdomen was tender, and there was a soft palpable liver. The patient developed seizure and apnea on the second day of admission; however, neck stiffness and Kernig’s sign were challenging to be assessed. After excluding the contraindications, a lumbar puncture was performed with a normal cerebrospinal fluid examination (CSF) and negative culture. The chest examination was shown to be normal, with normal CXR, and the ECG showed sinus tachycardia. The patient weighed 25 kg (on the 5th percentile), and his height was 140 cm (above the 50th percentile). He was kept in an isolated room with consideration of the possible diagnosis of SARS-CoV-2. Likewise, MIS-C was among the differential diagnosis. Therefore, SARS-CoV-2 IgG, and reverse transcription polymerase chain reaction (RT-PCR) COVID-19 were also performed.

Since the patient had a skin rash, vancomycin was rapidly switched to meropenem to avoid confusion with vancomycin-related rash, which is still common in our practice, in addition to ceftriaxone (50 mg/kg/dose) iv, every 12 h. Dexamethasone (0.15 mg/kg/dose) iv, every 6 h, and acyclovir (60 mg/kg/day) were started from the first day concerning the suspicion of meningitis or encephalitis. Diazepam and phenobarbital were administered due to the occurrence of seizures. A nasogastric tube was inserted for oral feeding to be resumed by the next day. Thus, he was kept on iv fluid, dexamethasone, antibiotics, acyclovir, and anticonvulsants. On the third day, with close monitoring, the multidisciplinary team concluded that MIS-C criteria were fulfilled according to the CDC/WHO definitions (Table 2) [7]. Therefore, iv methylprednisolone (M-PRD) was given in a dose of 25 mg twice a day (1 mg/kg/12 h) for 5 days, along with intravenous immunoglobulin (IVIG) infusion using (2 g/kg/day) given over 12 h for 2 days [10,11]. Subsequently, the patient showed striking improvement. The next day he regained consciousness, vital signs started to be stabilized, and the edema and redness were decreasing. Esomeprazole (10 mg once daily) was added on day three to avoid steroid-induced stomach irritation. Sinus tachycardia was detected since admission, and on the third day, mild dilation of the left ventricle and mild pericardial effusion were noticed through sequential ECG and echocardiography monitoring. Antithrombotic treatment with aspirin 81 mg orally was added on day six, and he carried on with oral prednisolone. Significant improvement was noticed after ambulation within a few days. Physiotherapy was part of his management. We continued alternate-day oral prednisolone for four weeks and aspirin for eight weeks with continuous monitoring until a normal quality of life was regained.

In summary, during the 10-day period of the patient’s stay in the hospital, he was deteriorating over the first three days in terms of fever, impaired level of consciousness, and the occurrence of seizure and apnea. Similarly, the laboratory data related to liver and renal function were deteriorating.

He was treated empirically as a case of toxic shock syndrome with antibiotics, and a glucocorticoid was given with consideration of central nervous system infection. But soon after receiving IVIG and M-PRD, he showed a substantial response. His consciousness improved by day 4, and GCS became (13/15), with less edema and good oral intake. Rising levels of (CRP, LDH, D-dimer, GGT, ALT, AST, s. ferritin, troponin T, and procalcitonin) were observed until the fifth day of admission. However, D-dimer and GGT did not stop increasing until the eight day. Meanwhile, according to the CBC, the blood parameters were gradually improving. Serial echocardiography revealed mild left-ventricular dysfunction. On day 9, he was back to normal clinically, and his liver enzymes, including GGT, were starting to decrease. By day 10, he had normal heart rates and echocardiography. D-dimer returned to normal in 1 month.

Of note, and perhaps from a social point of view, the parents hid the information that COVID-19 was confirmed in more than one family member four weeks before the onset of symptoms in the patient. Additionally, no one in the family, including the child, had received COVID-19 vaccination.

## 3. Discussion

It seems that few MIS-C cases were reported in the literature from Arab-Asian ethnicities, which does not mean a paucity of cases but rather a lack of documentation or even a lack of awareness, especially in developing countries [4,12]. Therefore, we sought to report the above case and highlight the subject to draw more attention to MIS-C among healthcare workers in our locality. To the best of our knowledge, it is the first case from Mosul, Iraq, to be reported. Our suggested approach was supported by the observational data in children from the growing literature concerned with MIS-C [2,3,4,5,6].

As the patient presented with impaired consciousness after blunt trauma, a head injury was to be excluded at the first step, especially concerning the nose bruises and bleeding. In addition, due to the presence of fever, persistent sinus tachycardia, tachypnea, skin rash, red eyes, impaired mental status, along with hypotension and oliguria, the toxic shock syndrome was expected from the first few minutes. On the other hand, central nervous system infection (i.e., meningitis or encephalitis) was considered in the differential diagnosis, especially after seizures. However, signs of meningeal irritation were challenging to be assessed. Meanwhile, there was no proven microbial infection, as the blood and CSF cultures were negative. Indeed, a seizure could also be related to trauma, and it might be encountered in 10% of children with head injuries [13]. The edema of his face could be due to the fall-associated bony injuries, but that was unlikely, as no bruises behind his ears or around his eyes (no raccoon eyes) were found, and his head CT was normal. Although the patient’s age was not in favor of Kawasaki disease, there were overlapping features highly suspicious of Kawasaki disease [6]. Other diseases, such as hemolytic uremic syndrome, thrombotic thrombocytopenic purpura, disseminated intravascular coagulation, hemophagocytic lymphohistiocytosis, systemic lupus erythematosus, vasculitis, appendicitis, cellulitis of the face, and other infectious diseases, were also in consideration and were excluded accordingly.

On the second day of admission, MIS-C was considered particularly when SARS-CoV-2 IgG was shown to be high while the RT-PCR of COVID-19 was negative, indicating a previous COVID-19 infection.

Furthermore, due to the overlapping features between COVID-19 acute infection and MIS-C, both need to be differentiated. Clinically, a previously healthy child, as in our case, with the absence of severe pulmonary involvement and the presence of mucocutaneous findings in addition to the cardiac and gastrointestinal manifestations, made severe acute COVID-19 an unlikely diagnosis. Interestingly, respiratory symptoms were commonly observed in adolescents with MIS-C, whereas conjunctival features, rash, and abdominal pain were more frequently encountered in younger children [5]. Moreover, regarding investigations, the inflammatory markers (CRP, ferritin, and D-dimer) tend to be much higher with MIS-C compared to severe acute COVID-19 [14]. It was also demonstrated that inflammatory markers were related to the severity of the MIS-C, and those children who were presented with shock had higher CRP levels and higher neutrophil counts when compared to those without shock, and that reduced counts of lymphocytes and platelets were associated with ICU admission and shock. Similarly, children with shock more commonly demonstrate high levels of cardiac markers [15,16]. All these findings were documented in our patient. The negative RT-PCR, while it excludes acute COVID-19 infection, is not against the diagnosis of MIS-C, as it is shown to be negative in 48% of MIS-C cases [5].

Remarkably, the family admitted, after the child recovered, that there was a COVID-19 documented RT-PCR infection in more than one member of the family 4 weeks before the appearance of the symptoms in our case; however, the latter had no symptoms at that time, and no test was performed for him.

Given that hospitalization rates for unvaccinated children were twice as high as for vaccinated children means that vaccines could effectively prevent morbidities associated with COVID-19 [17]. Unfortunately, the level of vaccination administered per 100 population in Iraq was quite low compared to the surrounding countries (excluding Syria), according to the WHO report of 9 August 2023 [18], which might be due to hesitancy and safety concerns.

Indeed, the collaboration of several specialty doctors, a neurosurgeon, a pediatric hematologist, a pediatric cardiologist, and an intensivist, along with the general pediatrician, improved the approach and management of our patient. It was shown that to minimize delays in recognizing MIS-C and its related morbidity and mortality, it is crucial to apply a multidisciplinary approach to improve early identification and treatment [4,19].

## 4. Conclusions

The 10-year-old Iraqi boy of Arab-Asian ethnicity was presented in a confusing neurosurgical/medical emergency mimicking a head injury overlapping the original presentation of MIS-C. Such a presentation and the severity of illness are a continuum that might be clinically difficult to distinguish. The stumble in obtaining an accurate history adds more delay in the diagnosis; therefore, open communication from parents to the healthcare providers no doubt helps avoid the repercussions.

This report highlighted the need to increase awareness about MIS-C among health workers in Iraq. Through the contribution of a multidisciplinary team, we reached the diagnosis, and therefore, we emphasize the need for such an approach in Iraq due to the importance of timely treatment.

Finally, vaccination should be encouraged in Iraq to prevent COVID-19-associated hospitalization and its related complications.

## Figures and Tables

**Table 1:** Laboratory results of the patient from Iraq with multisystem inflammatory syndrome in children (MIS-C) upon admission.

Tests		Values		Reference Ranges	Notes
CBC					
	Hb	9.6 g/dL	Low	11.5–15.5 g/dL	After 3 weeks, Hb returned to normal level
	Platelets	15 × 10^9^/L	Low	177–381 × 10^9^/L	After 3 weeks, platelets returned to normal level
	WBC	2.4 × 10^9^/L	Low	4.5–13.5 × 10^9^/L	On day 6, WBC returned to normal level
	Neutrophils	67%	High	54%	On day 4, started to improve
	Lymphocytes	29%	Low	38%	On day 4, started to improve
LFT					After 3 weeks, LFT returned to normal level
	ALT	212 U/L	High	7–55 U/L	
	AST	387 U/L	High	8–48 U/L	
	LDH	462 U/L	High	122–222 U/L	
	GGT	166 IU/L	High	8–61 U/L	
	ALP	212 U/L	High	40–129 U/L	
	Albumin	3.5 g/dL		3.5–5 g/dL	
	Bilirubin (total)	0.84 mg/dL		0.1–1.2 mg/dL	
RFT					After 3 weeks, RFT returned to normal level
	Creatinine	1.4 mg/dL	High	0.6–1.3 mg/dL	
	BUN	50 mg/dL	High	7–18 mg/dL	
Others					
	CRP	18.3 mg/L	High	8–10 mg/L	After 3 weeks, returned to normal level
	D-dimer	5120 ng/mL *	High	<500 ng/mL	Reached the peak at day 8, then after 4 weeks returned to normal level
	S. ferritin	400 ng/mL	High	7–140 ng/mL	
	Troponin T	0.44 ng/ml	High	<0.1 ng/ml	
	Procalcitonin	88.3 ng/mL	High	0.1 ng/mL	
	SARS-CoV-2 IgG	High	High	Negative	
	RT-PCR COVID-19	Negative		Negative	

* D-dimer result of day 8. ALP, alkaline phosphatase; ALT, alanine aminotransferase; AST, aspartate aminotransferase; BUN, blood urea nitrogen; CBC, complete blood count; CRP, c-reactive protein; GGT, gamma-glutamyl transferase; Hb, hemoglobin; LDH, lactate dehydrogenase; LFT, liver function test; RFT, renal function test; RT-PCR, reverse transcription polymerase chain reaction; SARS-CoV-2 IgG, severe acute respiratory syndrome coronavirus 2 immunoglobulin; WBC, white blood cells.

**Table 2:** Multisystem inflammatory syndrome in children (MIS-C) diagnosis in the case reported from Iraq according to CDC and WHO criteria.

	Variable	CDC Defined MIS-C *	WHO Defined MIS-C **	Our Patient (Fulfilled CDC and WHO Criteria)
1	Age (years)	<21	(0–19)	10
2	Fever	38.0 °C or more, or a subjective fever	For 3 days or more	Fever > 38 °C for 3 days
3	Multisystem involvement (at least 2 of the following):			
	A. Circulatory	Shock	Hypotension or shock	Shock
	B. Dermatology	Erythema or edema of hands or feet, oral mucositis, drying or fissuring of the lips, strawberry tongue, conjunctivitis, or other rash	Rash or bilateral non-purulent conjunctivitis or mucocutaneous inflammation (mouth, hands, or feet)	Rash, bilateral non-purulent conjunctivitis, drying and fissuring of the lips, strawberry tongue, and red edematous hands and feet
	C. Cardiovascular	Increased troponin or left ventricular ejection fraction < 55% or coronary artery dilation, aneurysm, or ectasia	Myocardial dysfunction, pericarditis, valvulitis, or coronary abnormalities (ECG changes or elevated troponin/BNP)	Increased troponin level and ECG changes
	D. Hematologic	Platelets < 150,000/μL or absolute lymphocyte count < 1000/μL	Evidence of coagulopathy (increasing D-dimer and prolonged PT or PTT)	Increasing D-dimer, platelets < 150,000/μL, and absolute lymphocyte count < 1000/μL
	E. Gastrointestinal	Vomiting or abdominal pain, or diarrhea	Vomiting, diarrhea, or abdominal pain	Vomiting and diarrhea,
4	Laboratory data (evidence of systemic inflammation)	CRP 3 mg/dL or more	E.g., elevated ESR, CRP, or procalcitonin	Elevated CRP, procalcitonin, ferritin, LDH, and neutrophils
5	SARS-CoV-2 infection (any of the mentioned points)	i. Confirmation by:	Evidence through:	There was evidence of:
	-RT-PCR	a. Positive RNA (e.g., RT-PCR) during hospitalization or within 60 days prior to or postmortem	a. Positive SARS-CoV-2 RT-PCR	Negative SARS-CoV-2 RT-PCR
	-Serology	b. Positive antibodies (serology) associated with current illness	b. Positive serology	Positive serology
	-Antigen test	c. Positive antigen test during hospitalization or within 60 days prior or postmortem	c. Positive antigen test	Not conducted
		or		
	-History of contact	ii. Evidence of exposure (close contact with a confirmed or probable COVID-19 case within 60 days before hospitalization)	d. Contact with a patient with COVID-19	Contact with a patient with COVID-19
6	Absence of	A more likely alternative diagnosis	Obvious microbial causes of inflammation, including bacterial sepsis and staphylococcal/streptococcal toxic shock syndromes	No alternative plausible diagnoses and no obvious microbial cause of inflammation, including bacterial sepsis and staphylococcal/streptococcal toxic shock syndromes, were detected
7	General health status	Critical and in need of emergency setting/or resulting in death		Critical and required emergency setting

BNP, brain natriuretic peptide; CDC, Centers for Disease Control and Prevention; COVID-19, coronavirus disease; CRP, C-reactive protein; ESR, erythrocyte sedimentation rate; PT, prothrombin time; PTT, partial prothrombin time; RNA, ribonucleic acid; RT-PCR, reverse transcription polymerase chain reaction; SARS-CoV-2, severe acute respiratory syndrome coronavirus 2; WHO, World Health Organization. * All 7 criteria should be met in the CDC definition. ** All 6 criteria should be met in the WHO definition.

## Data Availability

The data that support the findings of this study are available from the corresponding author, Lika’a Fasih Y. Al-Kzayer, upon reasonable request.
